# Sequence analysis and pathogenicity of Avian Orthoavulavirus 1 strains isolated from poultry flocks during 2015–2019

**DOI:** 10.1186/s12917-020-02470-9

**Published:** 2020-07-22

**Authors:** Hatem S. Abd El-Hamid, Manal E. Shafi, Najah M. Albaqami, Hany F. Ellakany, Naglaa M. Abdelaziz, Mohamed N. Abdelaziz, Mohamed E. Abd El-Hack, Ayman E. Taha, Khalid M. Alanazi, Ahmed R. Elbestawy

**Affiliations:** 1 Department of Poultry and Fish Diseases, Faculty of Veterinary Medicine, Damanhour University, Damanhour, El Beheira 22511 Egypt; 2 Department of Biological Sciences, Zoology, King Abdulaziz University, Jeddah, 21589 Saudi Arabia; 3 Reference Laboratory for veterinary Quality control on Poultry production (RLQP), Animal Health Research Institute, ARC, El Dokky, Giza, Egypt; 4 Department of Poultry, Faculty of Agriculture, Zagazig University, Zagazig, 44511 Egypt; 5 Department of Animal Husbandry and Animal Wealth Development, Faculty of Veterinary Medicine, Alexandria University, Edfina, 22758 Egypt; 6 Zoology Department, College of Science, King Saud University, P.O. Box 2455, Riyadh, 11451 Saudi Arabia

**Keywords:** Avian Orthoavulavirus 1, F protein, Sequencing, Amino acid residue substitution, Intracerebral

## Abstract

**Background:**

Newcastle disease (ND) causes severe economic losses in poultry industry worldwide. Egyptian poultry industry suffered from severe economic losses since the isolation of Velogenic Newcastle disease virus (vNDV) genotype VIId in 2011 and up till now despite the use of different vaccination programs. So, this study aimed to isolate and characterize the vNDV from a total of 120 poultry flocks from ten provinces in the Egyptian Delta region with a history of respiratory manifestation, high mortalities or a decrease in egg production between 2015 and 2019. Seventy-three samples’ allantoic fluid (73/120, 60.8%) were positive for hemagglutination with chicken RBCs. These samples were submitted to molecular examination using qRT-PCR specific primers for AOAV-1, highly pathogenic avian influenza (HPAI-H5), low pathogenic avian influenza (LPAI-H9) and infectious bronchitis virus (IBV).

**Results:**

Fifty samples (50/120: 41.6%) were confirmed positive for AOAV-1, based on genetic analysis of matrix and fusion protein. The co-infection rate of other respiratory viral diseases examined was 1.6, 14.1, and 4.1%, for HPAI-H5, LPAI-H9, and IBV, respectively. Biologically, the intracerebral pathogenicity index of ten selected AOAV-1 isolates ranged from 1.70 to 1.98, which indicated the velogenic nature of these isolates. All the sixteen sequenced isolates were AOAV-1 genotype VII.1.1. The full F gene sequence of six examined AOAV-1 VII.1.1 isolates contained the seven neutralizing epitopes, and the glycosylation motif of six-potential sites for N linked glycosylation at residues 85, 191, 366, 447, 471, and 541.

**Conclusion:**

It could be concluded that the high prevalence of AOAV-1 genotype VII.1.1 in the Egyptian chicken flocks despite the intensive vaccination with live and killed ND vaccines, as all the 16 isolates tested were belonged to this genotype. Homologous vaccination is badly needed to control and reduce the spread of AOAV-1 genotype VII.1.1infection in Egyptian poultry flocks.

## Background

Newcastle disease (ND) encompasses a critical impact amongst the most economically viral poultry diseases leading to high mortality and morbidity rates in the susceptible poultry. It still causing a drop in egg production even in vaccinated layers [1]. Besides the economic impact through the loss of productive assets, trade restrictions are of significant concern for exporting countries. Within the last 5 years, 109 of 200 member countries have reported the disease to the OIE [2].

Newcastle disease virus (NDV) or Avian paramyxoviruses 1 (APMV-1) was recently classified to Genus Avian Orthoavulavirus, family Paramyxoviridae and common NDV is recently known as Avian Orthoavulavirus-1 (AOAV-1) [3, 4]. Full fusion (F) gene nucleotide sequencing classified all AOAV-1 into two classes; class I and class II. AOAV-1 class II contains virulent, and non-virulent viruses, with 21 identified genotypes (I to XXI Genotype). Updated classification criteria of AOAV-1 genotype VII viruses merged all the subgenotypes into only 3, VII.1.1 including previous subgenotypes (b, d, e, j, l), VII.1.2 included previous subgenotypes (f), and VII.2 included the previous subgenotypes (h, i, k) [5].

AOAV-1 (genotype VII) causes fatal infections in poultry and other susceptible birds as they are responsible for the fourth major panzootic of ND worldwide which caused by viruses of genotype VII.1.1 [6]. Similarly, in the Egyptian poultry industry, AOAV-1 is specially subgenotypes VIId and VIIb are incriminated in significant economic losses. The partial sequence of the F gene and phylogenetic analysis of AOAV-1 isolated from different outbreaks in Egypt from 2011 to 2017 belonged to genotype VIId [7–10].

Among genotype VII viruses, sub-genotype VIIi has demonstrated an intercontinental spread and, therefore, has a global significance in the perspective of potential fifth panzootic [6]. Most of the AOAV-1 viruses that are pathogenic for chickens have the sequence 112R/K-R-Q/K/R-K/R-R116 at the C-terminus of the F2 protein and F (phenylalanine) at residue 117 at the N-terminus of the F1 protein. In contrast, the viruses of low virulence have sequences in the same region of 112G/E-K/R-Q-G/E-R116 and L (leucine) at residue 117 [11, 12]. Thus, there is a need for at least one pair of basic amino acids at residues 116 and 115 plus phenylalanine at residue 117 and a basic amino acid (R) at 113 to enable the virus to show virulence for chickens.

Study of F protein full length during outbreaks of 2016 in poultry flocks in Egyptian provinces revealed that genotype VIId viruses of class II are the most predominant AOAV-1 stains [13]. The phylogenic analysis of AOAV-1 strains circulated in small-scale poultry holdings in Egyptian villages revealed them belonging to genotype VIIb. Also, the viral transmission occurred among neighboring farms over long distances, and co-infections with multiple pathogens were identified [14, 15]. Circulating AOAV-1 in wild birds in borderline or frontier Egyptian provinces containing wetlands was recorded [16]. Consequently, this study aimed to investigate the molecular and biological characterization of the AOAV-1 strains from 2015 through 2019 in the Egyptian Delta region provinces.

## Results

### Isolation of AOAV-1 in SPF-ECE and slide HA testing

The embryos of inoculated eggs were died between the 2nd and the 4th days post-inoculation in many samples. Samples with no mortality after the 3rd passage that had no HA activity were confirmed as negative for haemagglutinating viruses. A total of 73 samples were positive with slide HA test constituting 60.8% of total AF samples (Table 1). The percent of samples had positive HA test from different provinces were as follows: El Beheira 58.7%, Alexandria 76.9%, El Gharbia 53.3%, El Qaliobia 44.4%, El Dakahlia 57.1%, Kafer El Shiekh 66.6%, El Menofia 66.6%, El Giza 100%, Marsa Mattrouh 50% and El Sharkia 100%.

**Table 1:** The HA, qRT-PCR confirmation of AOAV-1 with other viruses in the Egyptian provinces of Delta Region

	Province	Total samples / province	HA negative	HA positive	Total positive samples for examined viruses using qRT_PCR			
					Positive AOAV-1/total samples of each province	H5 positive samples	H9 positive samples	IBV positive samples
Total Samples 120	El Behiera	63	26	37	26/63: 41.2%	0	11	2
	Alexandria	13	3	10	4/13: 30.7%	0	4	1
	El Gharbia	15	7	8	8/15: 53.3%	1	0	0
	El Qaliobia	9	5	4	2/9: 22.2%	0	0	–
	El Dakahlia	7	3	4	3/7: 42.8%	0	0	–
	Kafr El Shiekh	3	1	2	2/3: 66.6%	0	0	–
	EL Menofia	3	1	2	2/3: 66.6%	0	0	–
	El Giza	3	0	3	0/0: 0%	1	2	1
	Marsa Mattrouh	2	1	1	1/2: 50%	0	0	–
	El Sharkia	2	0	2	1/2: 50%	0	0	1
Total	10	120	47	73	50/120: 41.6%	2/120: 1.6%	17/120: 14.1%	5/120: 4.1%

ND + H9: 2; ND + H5: 1; ND + IB: 2; ND + H9 + IB: 1 and 7 haemagglutinating samples were negative for all examined viruses

### The prevalence of AOAV-I and other viruses in provinces of the Egyptian Delta region

Fifty AOAV-1 isolates (50/120: 41.6%) were confirmed, 2/120 isolates (1.6%) were positive for HPAI-H5N1, 17/120 (14.1%) isolates were positive LPAI-H9N2 and 5/120 (4.1%) were IBV positive. The mixed infection of AOAV-1 with H9N2 appeared in two samples. One positive AOAV-1 sample was mixed with H5N1 infection. Two samples of AOAV-1 were mixed with IBV. One AOAV-1 sample was mixed with both H9N2 and IBV. From all the 73 HA positive samples, only seven samples were negative for all of the four viruses under study. The prevalence of virulent AOAV-1 remains the highest higher in the broiler farms in the Delta region representing 41.1% (37/90 flocks) of AOAV-1 isolates, followed by layer flocks 5/13 (38.4%). Despite the lower examined flocks of Balady, Sasso chickens, and pigeons; the confirmed AOAV-1 was high as 5/7 (71.4%) in Balady chickens; 1/3 (33.3%) Sasso chickens and 2/4 (50%) in pigeons. According to the geographical order: incidence of AOAV-1 in the ten Egyptian province farms was as follows: El Beheira 41.2%, Alexandria 30.7%, El Gharbia 53.3%, El Qaliobia 22.2%, El Dakahlia 42.8%, Kafer El Shiekh 66.6%, El Menofia 66.6% and El Giza 0% Marsa Mattrouh 50% and El Sharkia 50% (Table 1).

### Biological evaluation of the pathogenicity of AOAV-1 field isolates

Testing only ten AOAV-1 isolates (representing six Egyptian provinces) by ICPI gave values of 1.70–1.98 for all the ten isolates, which indicates the velogenic nature of these isolates (Table 2).

**Table 2:** Intra-cerebral Pathogenicity Testing for 10 selected AOAV-1 isolates and molecular, genetic pathotyping and accession numbers of all 16 (full and partial F gene sequenced AOAV-1)

Strain code	Province	Type of bird /age	Year of isolation	ICPI value	pathotype	Virulence Cleavage site motif	Genotyping	Accession number
1	El Behiera	Broiler	2015	n.d.	Velogenic	112RRQKRF117	VII.1.1	MH445410
2			2016	1.83				MK984289
3			2017	1.75				MN519688
4				1.83				MK984236
5		Broilers	2018	1.98				MK984237
6	Alexandria			1.92				MN519690
7	El Dakahlia			1.87				MN519692
8	Marsa Mattrouh			1.70				MN519696
9	El Sharkia			1.75				MN519693
10	El Behiera			1.86				MK984238
11	El Gharbia	Layer pullets		1.71				MN519689
12	Kafer Elshiekh	Layers		n.d.				MN519694
13	El Qaliobia	Broilers		n.d.				MN519687
14	Alexandria	Pigeons		n.d.				MN519685
15				n.d.				MN519686
16	El Behiera	Broilers	2019	n.d.				MN519684

*Abbreviation*: *n.d.* not done

### Sequence analysis of fusion protein, phylogenetic tree and molecular pathotyping of AOAV-1 isolates

The obtained full-length F protein nucleotides sequences of six AOAV-1 isolates submitted for BLASTN analysis revealed 99.2% identity with NDV-Egy-Beh-ck-VII-b-2016-NR726 and NDV-EG-35-2014 AOAV-1 isolates’ fusion (F) protein. Their GenBank accession numbers were as follows: MN519684, MK984236, MK984237, MK984239, MK984238, and MH445410. While GenBank accession number of the submitted partial fusion (F) protein sequences of the other ten AOAV-1 isolates were MN519689, MN519690, MN519691, MN519692, MN519693, MN519694, MN519685, MN519686, MN519687, and MN519688.

**Fig. 1:**
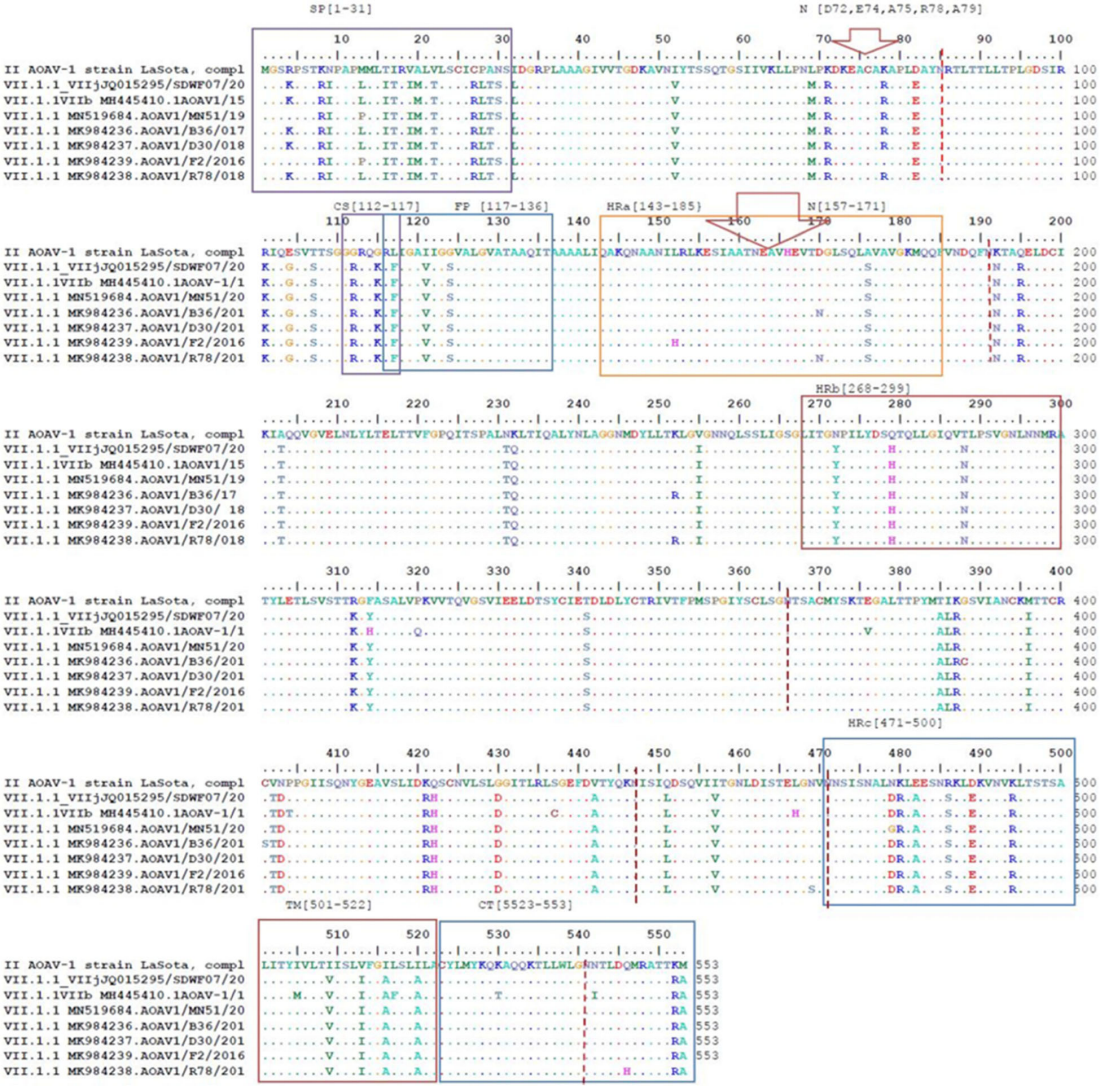
Alignment of deduced amino acid of full fusion protein of 6 isolates under study and consensus reference, Egyptian and vaccinal strains**.** SP = signal peptide, CS = Cleavage site, FP = Fusion Peptides, HR = Heptad repeats (a,b,c),TM = transmembrane domain, CT = cytoplasmic tail, the arrow refers to the neutralizing epitopes and strip refers to 6 glycosylation sites

**Fig. 2:**
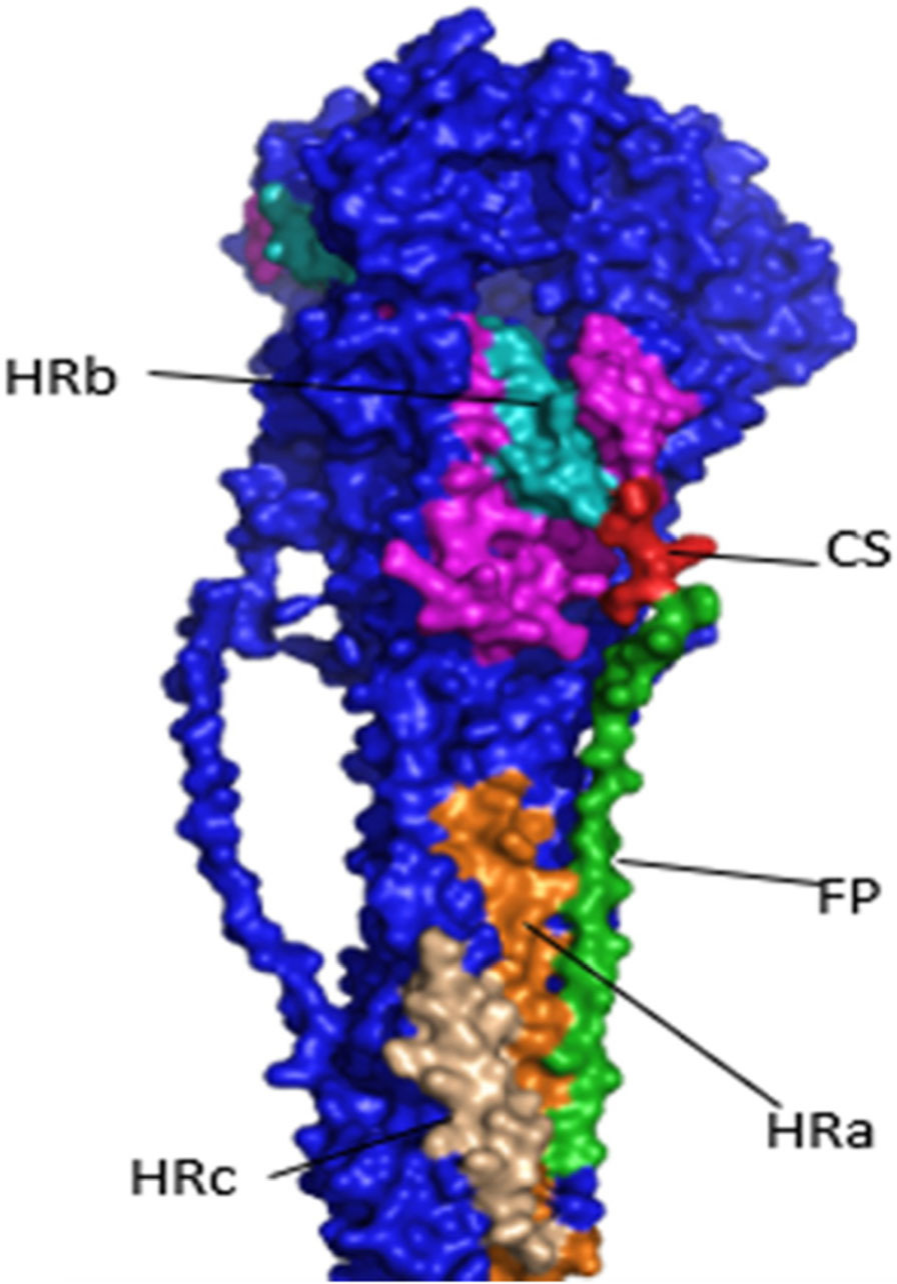
3D structural of fusion protein of AOAV-I VII.1.1 by Paymol

**Fig. 3:**
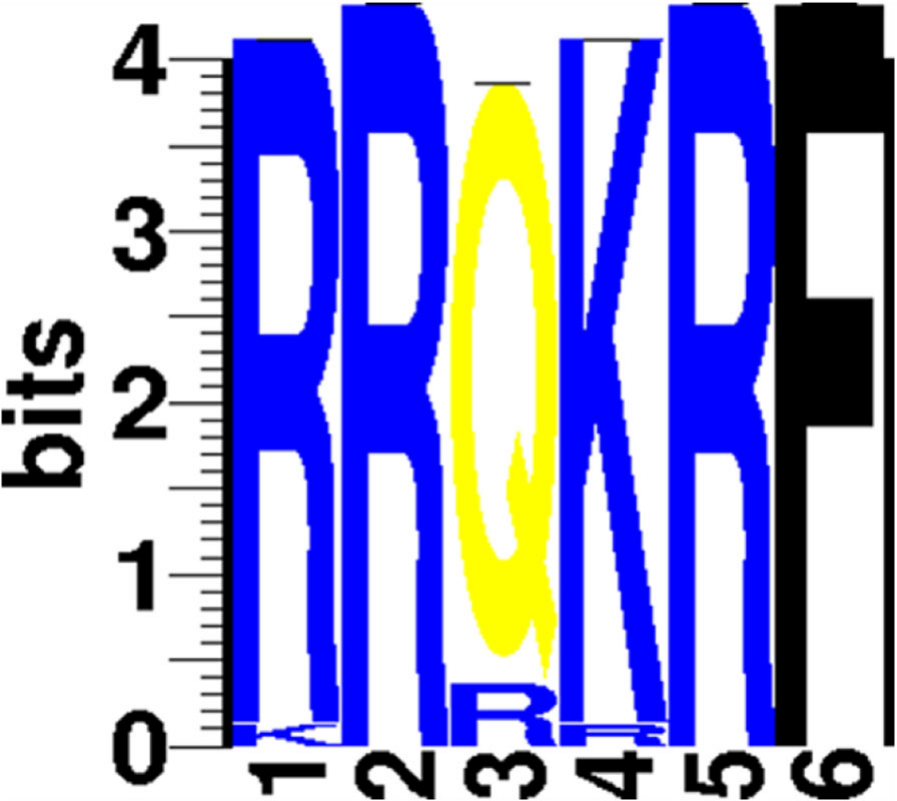
Sequence logo (SeqLogo) generated from amino acid sequence alignment which is a useful tool to visualize sequence patterns and represent a more informative alternative to consensus sequence

The comparative alignment of the deduced amino acid of the first six AOAV-1 isolates confirmed the presence of multiple basic amino acids at the positions of 112 to 116 and F phenylalanine aa at the position of 117 (the cleavage site motif of virulent strains RRQKRF) along with the conserved amino acids of K101 and V121. Both are characters of more virulent AOAV-1 viruses confirming the velogenic pathotyping of these isolates (Table 3 and Figs. 1, 2 and 3).

**Table 3:** Amino acids substitutions in function domain of F protein

ID Fusion F0 protein (1–553) aa	Signal peptide SP aa (1–31)	Fusion F2 subunit aa (32–116)		Fusion F1 subunit (117–533) aa					
		(32–111aa)	Cleavage site CS aa (112–117)	Fusion peptides FP (117–136) aa	Heptad repeat HRa (143–185) aa	Heptad repeat HRb (268–299) aa	Heptad repeat HRc (471–500) aa	Transmembrane TM (501–522) aa	Cytoplasmic tail CT (523–553) aa
	30S	78R	112–117aa	–	152 L, 170D	–	479D	505I, 517 L	531A, 541 N, 546Q
MN519684 (AOAV-Eg-Ch-MN51–2019, F, Complete)	S	R	RRQKRF	–	–	–	479G	–	–
MK984236 (AOAV1-Eg-Ch-B36–2017, F, Complete)	N	R	RRQKRF	–	- 170 N	–	–	–	–
MK984237 (AOAV1-Eg-Ch-D30–2018, F, Complete)	N	R	RRQKRF	–	–	–	–	–	–
MK984239 (AOAV-Eg-Ch-F2–2016, F, Complete)	S	K	RRQKRF	–	152H -	–	–	–	–
MK984238 (AOAV1-Eg-Ch-R78–2018, F, Complete)	N	R	RRQKRF	–	- 170 N	–	–	–	546H
MH445410.1 (AOAV1-Eg-Ch-18-2015, F, Complete)	S	R	RRQKRF	–	–	–	–	505 M, 517F	531 T, 541I

**Fig. 4:**
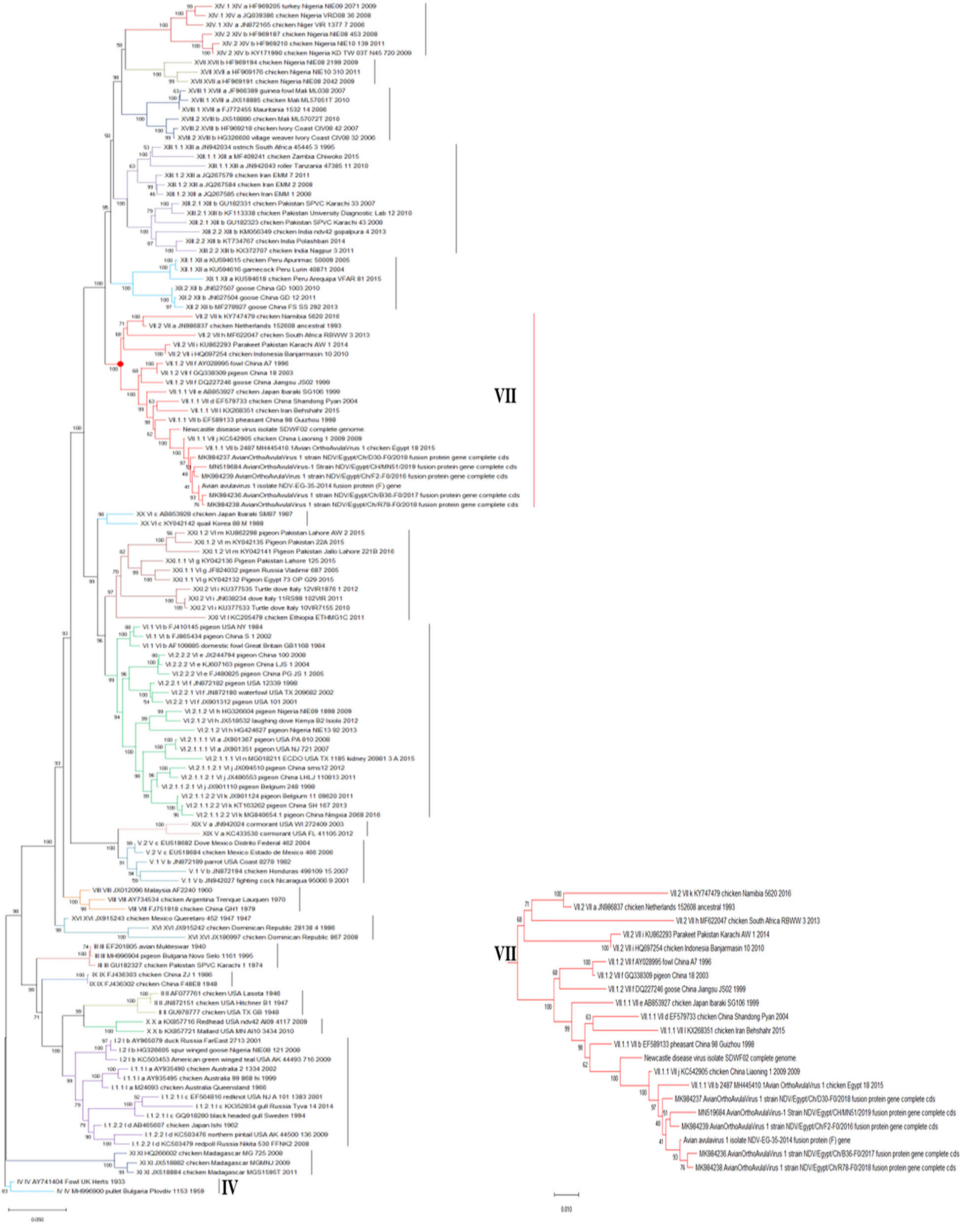
Phylogenetic tree involved nucleotide sequence represent all genotypes of AOAV-1 (From I to XXI) and branch red color represent Genotype VII and the 6 isolates arranged with genotype VII. evolutionary analysis by Maximum Likelihood method. This analysis involved 127 nucleotide sequences. There were a total of 1659 positions in the final dataset

**Fig. 5:**
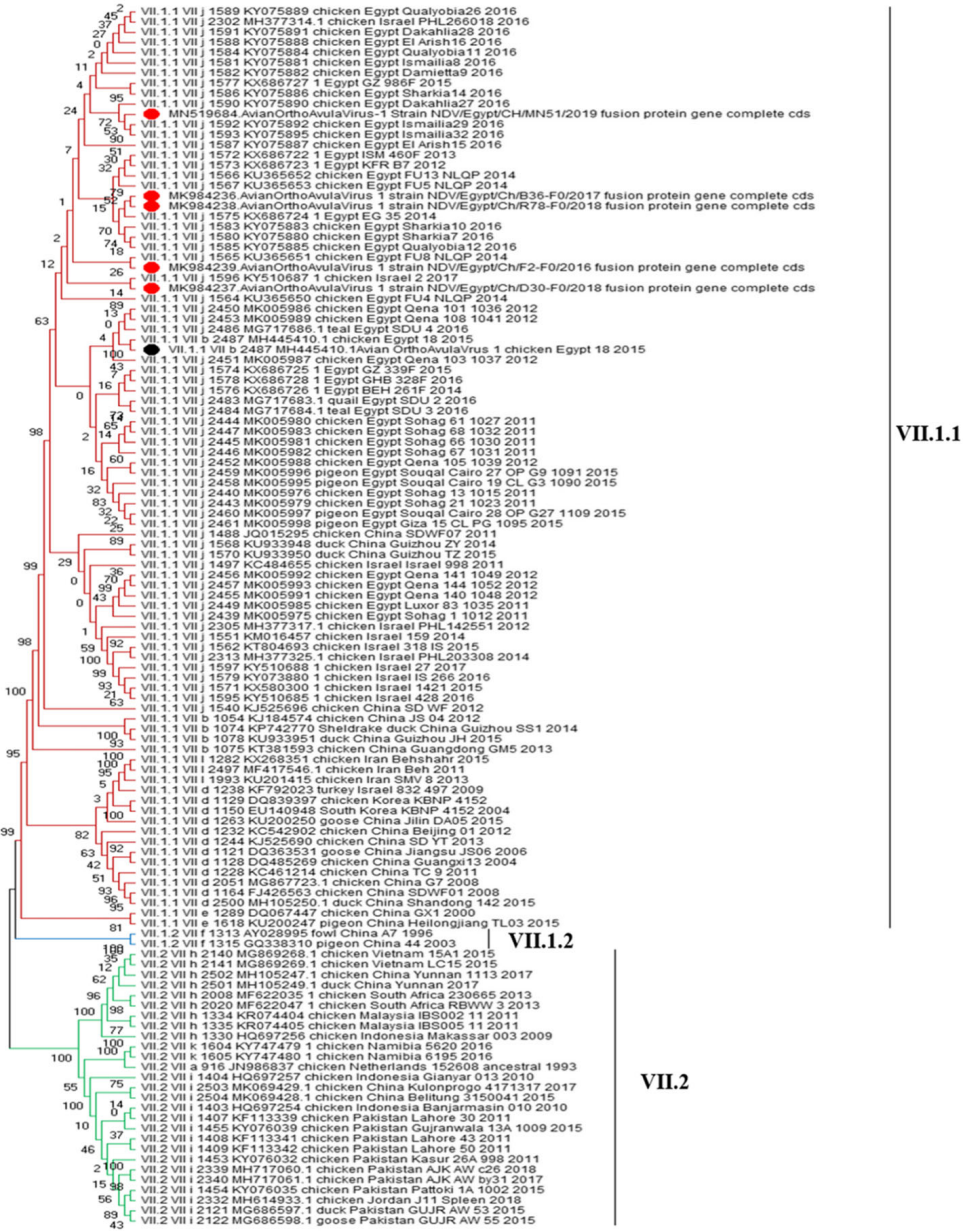
Evolutionary analysis by Maximum Likelihood method. The tree is drawn to scale, with branch lengths measured in the number of substitutions per site. This analysis involved 118 nucleotide sequences. Codon positions included were 1st, 2nd, 3rd, noncoding. There were a total of 1660 positions in the final dataset. Red solids refer to 6 isolates of the study

All these six AOAV-1 isolates from 2015 to 2019 submitted for full F genomic sequencing were recognized as AOAV-1 genotype VII.1.1. (Fig. 4) based on the complete F protein and according to the classification of Dimitrov et al. [5]. Those six AOAV-1 isolates were clustered with subgenotype VIIj except of the isolate MH445410 which was clustered in the subgenotype VIIb (Fig. 5). Also, the other ten AOAV-1 isolates partially sequenced for the F gene were also clustered with the subgenotype VIIj according to Diel et al. [17, 18].

The nucleotides alignment of the complete F protein sequence (1–1659) and the phylogenetic tree of the six isolates confirmed the high similarity and close relatedness by 98.1–99.3% to the Egyptian isolates from 2011 to 2015 and by 97.4–99.3% identity to the Egyptian isolates from 2016 to 2019. Also, these six isolates showed a high similarity (98.3–99.1%) with the other Middle East strains of genotype VII as well as with the Chinese strains, and with a lower identity of 94.1–95.1% with the Korean strains.

**Fig. 6:**
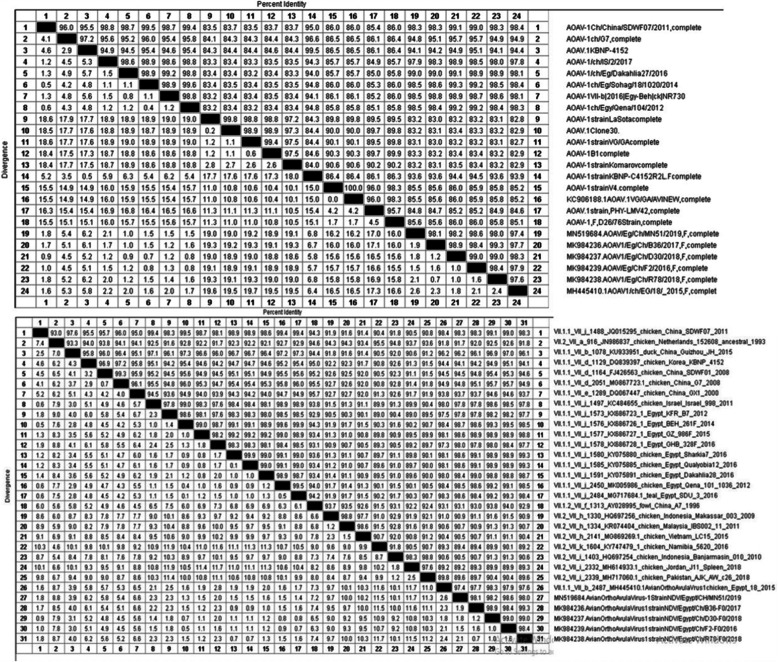
Nucleotide identity and divergence percent between 6 AOAV-1 genotypes VII.1.1 and consensus global, Egyptian and vaccine strains

Regarding the nucleotide identity between those six AOAV-1 genotypes, VII.1.1 isolates and the vaccine strains used in the prevention and control of the AOAV-1 infection in Egypt was so far, and this nucleotide identity varied from one vaccine strain to another. For class II, genotype II vaccine strains, an 82.8–83.2% identity was recorded with LaSota, 82.9–83.4% with both Clone 30 and Hitchner B1, and 85.2–86% with VG/GA (Avinew®). While the relatedness between our six isolates and the genotype I vaccine PhyLM42 strains were 84.6–85.2% with V4 which was 85.2–86%, and with D26/76 which was 85.1–86%. Whilst, the relatedness of these six isolates with genotype VII vaccine (KBNPC415R2L) F gene had higher nucleotide identity that ranged from 93.6 to 94.5%, indicating a closer genetic relationship (Fig. 6).

### Antigenic analysis of the AOAV-1 isolates

All the AOAV-1 genotypes VII.1.1 isolates contained the seven neutralizing epitopes which are essential for the function of the fusion protein, the conserved amino acid at position D72, E74, A75, R78, A79, 157SIAATNEAVHEVT-171, and L343. Only the isolate designated MK984239 (AOAV-Eg-Ch-F2–2016) had an amino acid substitution in position R78K. Also, all the six isolates contained the conserved amino acids at Domains III and I at positions of I196, I274, D277, Q286, V287, P290, L295 and N297 which are important for folding and fusion activity and contained the conserved ten amino acid cysteine sites at positions C76, C199, C338, C347, C362, C370, C394, C399, C401, C424 except isolate MK984236 (AOAV1-Eg-Ch-B36–2017), contains the amino acid substitution at position C401S to serine. Glycosylation motif of all the six isolates revealed that the six-potential sites for N linked glycosylation are at residues 85, 191, 366, 447, 471, 541 shown as a red strip in Fig. 1.

Also, the deduced amino acid substitutions were identified in the six AOAV-1 isolates of genotype VII.1.1, were as follows: in the isolate MN519684 (AOAV-Eg-Ch-MN51–2019) complete replacement occurred in signal peptide region at positions R4K, L13P and in F1 Heptad repeat c - HRc (471–500 aa), a substitution was in the position of D479G. While the isolate designated, MH445410 (AOAV1-ch-EG-18-2015), had a substitution that took place in the signal peptide region in position of K19V. In the other three isolates, no. MK984236 (AOAV1-Eg-Ch-B36–2017), MK984237 (AOAV1-Eg-Ch-D30–2018), and MK984238 (AOAV1-Eg-Ch-R78–2018), a substitution occurred in signal peptide region at position S30N sharing the same amino acid N30 of genotype II of the vaccinal strains.

The HRa region in MK984236 (AOAV1-Eg-Ch-B36–2017) had a substitution in the position of D170N, and this isolate also had a substitution at positions K252R, G308C, and conserved C401S. The isolate no. MK984239 (AOAV-Eg-Ch-F2–2016) had a complete substitution in the HRa region in position L152H. Meanwhile, in the isolate no. MK984238 (AOAV1-Eg-Ch-R78–2018), a complete substitution occurred in the HRa region at the position of D170N and another substitution at the positions of K252R, N469S, and N546H in the cytoplasmic tail. Finally, in the isolate MH445410 (AOAV1-ch-EG-18-2015), there was a substitution at the positions of Y314H, P320Q, E376V, P405T, S436C, L467H and I505 M, L517F of the transmembrane region and N531 T, A541I of the cytoplasmic tail region. Cytoplasmic tail important amino acids at positions 548 and 549 were conserved in all isolates and homologous to the vaccine strain (s).

## Discussion

Newcastle Disease (ND) or AOAV-1 is a highly contagious viral disease that affects more than 240 domestic and wild bird species, with a higher incidence in domestic poultry, resulting in severe outbreaks worldwide. It represents a major depletion in the poultry economy than any other viral disease [5, 19]. To date, the disease occurs on at least six of the seven continents [6], being an enzootic in many countries, with variable outcomes and subsequently variable economic losses.

Class II of AOAV-1 is more diverse, containing virulent and non-virulent viruses, and the complete analyses identified at least 21 distinct genotypes [5]. Genotype VII of the class II viruses had been associated with the most recent outbreaks in Asia, Europe, Africa, Middle East, and South America [20–24]. Genotype VII can be further divided into 12 (VIIa–l) subgenotypes according to amino acid substitutions [5, 25].

The previous studies regarding the partial sequence of F protein and phylogenetic analysis of AOAV-1 in the Egyptian poultry flocks revealed that the predominant strain circulating in the poultry field was viruses of sub-genotypes b and d [13–16, 26, 27]. The clinical manifestations and PM lesions of ND caused by the confirmed AOAV-1 in the examined poultry flocks as respiratory manifestations, enteric symptoms (greenish diarrhea) and nervous manifestation with high mortalities and decreased egg production and quality [amorphous (oddly shaped), smaller eggs with rough, thin, and whiter bleached shells and poor-quality contents whites and yolks] were similar to those findings of Miller and Koch [5], Terregino and Capua [28], Susta et al. [29], Bertran et al. [30] and Igwe et al. [31].

Herein, a total of 50/120 (41.6%) samples were positive for AOAV-I, indicating the higher incidence rate of this virus in the Egyptian poultry flocks. The higher rate of isolation of AOAV-1 from infected flocks collected during the winter season in the Delta region may be attributed to increasing the environmental load of AOAV-1 through the high viral stability during the cold and wet season, with increased poultry stocking density during winter. The higher air spreading of the virus for long distances with the help of wind and the transportation of untreated litter from broilers and layer hens to the agricultural fields for using as a fertilizer. The virus survives up to several months in the infected litter [32, 33]. The high density in poultry farms near backyard flocks facilitates the transmission of AOAV-1 [34].

Wang et al. [35] studied the risk factors of infectious diseases for poultry in backyard household flocks in rural China. Authors found that they were significantly affected by neighboring commercial poultry and close contact with wild birds. The role of both free-living pigeons in the villages nearby the chicken farms as well as ducks reared in house backyards can’t be neglected as a risk factor for chickens in the commercial sector for the NDV infection as most of the sampled farms have open house ventilation directly exposed to the outer environmental air draughts and dust. Artois et al. [36] investigated the risk factors of Avian Influenza in the Northern Egyptian Delta Governorates. They documented that the probability of HPAI (H5N1) presence was associated with a risk factor of the existence of duck farms with significant mortality or drop in egg production in the village near the chicken commercial farms.

Two previously published papers showed the ability of pigeons and ducks in transmitting this virus to chickens. The first study by Ellakany et al. [37] confirmed that in-contact chickens to infected pigeons developed severe respiratory, digestive and nervous signs and suffered from the mortality of 2/5 and 3/5 in the in-contact chickens to pigeons infected IM (intra-muscularly) and IN (intra-nasally) respectively. In-contact chickens to infected pigeons, either IM or IN showed high viral shedding titers in both oropharynx and cloaca up to 16 days post-exposure to the infected pigeons. From these results, it could be concluded that free-range pigeons can be considered as an efficient reservoir for NDV-VIId to opened-house raised broilers. A similar experiment by Elbestawy et al. [38] proved that Muscovy ducks infected with the velogenic (AOAV-1) NDV genotype VIId, by the intra-nasal route, shed the virus in their tracheas for up to 16 days PI and their in-contact infected chickens showed clear characteristic signs for NDV and 20% mortality. These in-contact chickens were still shedding the virus in their oropharyngeal and cloacal excreta for up to 11 days post-exposure to the infected ducks. This proved that Muscovy ducks are efficient carriers and considered as a risk factor for commercial chickens for infection with velogenic AOAV-1 -genotype VIId under the conditions of raising poultry in Egyptian villages. Otim et al. [39] evaluated the risk factors for Newcastle disease in Uganda villages. They found that purchasing restocking chickens from the market and neighborhood (hazard ratio [HR] = 1.79), the presence of migratory wild birds (HR = 1.70), and being in agro-ecological zone 1 (HR = 1.48) showed a positive but non-significant association with the risk for ND.

According to the OIE [2], NDV virulent strains (mesogenic and velogenic) possess 112R/K-R-Q-R/K-R-F117 motif with two pairs of basic amino acids and a Phenylalanine in the N terminus of the F1 (residue 117). The second OIE accepted criteria for virulent NDVs is the ICPI, in one-day-old chicks [5, 40]. AOAV-1 isolates were classified as virulent (mesogenic or velogenic) pathotype when the ICPI score was equal or greater than 0.7 [2]. In this study, the ICPI value for ten confirmed AOAV-1 isolates gave an ICPI score between 1.70 and 1.98 for all the isolates indicating their virulent pathotype nature. Previously, the ICPI values of most Egyptian AOAV-1 genotype VII viruses were recorded to be between 1.66 and 1.97 [9, 16, 27, 41, 42]. This increase in the ICPI score for the Egyptian isolates of AOAV-1 over the last 4 years indicates an increase in their virulence.

Regarding the molecular pathotyping of the other six selected isolates of AOAV-1 chosen for complete F gene sequencing, the comparative alignment of deduced amino acid revealed the presence of two pairs of multiple basic amino acids R/K at positions of 112 to 116 and F phenylalanine aa at position 117 which is the cleavage site motif of virulent strain RRQKRF. Also, the comparative alignments of all cleavage site motifs revealed that these six virulent strains contained the conserved amino acids at positions K101 and V121 of the fusion (F) protein which is the unique features of genotype VII) which agreed with the previous study of Lien et al. [43]. The presence of Q in the motif of virulent strain RRQKRF enhances and increases the virulence [44]. The full-length F gene sequencing of six and the partial sequencing of the other ten AOAV-1 isolates, respectively, showed 97.4–99.3% identity with the previously published Egyptian isolates between 2011 and 2019. This indicates the high prevalence of the AOAV-1 genotype VII.1.1 in the Egyptian poultry flocks.

Another test for virulence detection is the F protein sequence analysis of AOAV-1 which is a class I integral membrane protein present as a trimer in the virion. Initially, F0 is synthesized as an inactive precursor and subsequently cleaved by the cellular proteases into a disulfide-linked F1-F2 complex. The deduced amino acid of the functional domain of F protein of AOAV-1 contains signal peptide from position (1–31 aa), F2 subunit from position (32–116aa) contains the cleavage site (112aa to 116aa). The F1 subunit contains the fusion peptide FP from position (117 aa to 136aa), three hydrophobic heptad repeat (HR) domains (HRa 143aa-185aa, HRb 268aa − 299 aa and HRc 471aa − 500 aa), the transmembrane (TM) domain from (501 aa to 522aa) and cytoplasmic tail (523 to 553 aa) are important for viral infectivity and pathogenicity [45]. The amino acid substitution in neutralizing sites of F protein, especially substitutions in fusion peptides and hydrophobic regions and transmembrane regions, had alteration effect on the fusion activity. It helped the virulent virus to escape neutralizing antibodies as a variant virus [46–48]. Also, cytoplasmic tails had an importance for virus replication and vaccine competence [49].

All glycosylation sites were conserved in all tested isolates, which have an important role in the infectivity and antigenicity [50]. A signal peptide region substitution occurred in three isolates MK984236, MK984237, and MK984238 in the position of S30N sharing the same amino acid N30 of genotype II of vaccinal strains. This was previously studied in the Egyptian strain of genotype VIId [13], suggesting that the use of genotype II vaccine type in control of AOAV1 genotype VII.1.1 in Egypt may be implicated in the emergence of new variants rather than providing benefits against NDV infections. The HRa region in MK984236, and MK984238 had a substitution in the position of D170N that differ from conserved amino acid of neutralizing epitope strain MK984239. The same results were obtained by Selim et al. [27] who examined seven Egyptian strains of genotype VIId and found alterations at different sites of the F gene interest as N-glycosylation sites, epitopes binding sites cysteine residues that may affect virus pathogenicity and may interfere with the classical vaccine’s protection. Isolate no. MH445410 had the highest amino acid substitutions rate.

All signal peptides of the six tested AOAV-1 genotype VII.1.1 isolates contained the amino acid at positions of I9, A11, R18, C25 and D170 except for the two strains MK984236 and MK984238, which had substitution at position D170N sharing these amino acids characteristic for subgenotypes (VII b, d, e, g), that represent viruses of 4th panzootic, while the 5th panzootic of AOAV-1 infections were belonged to viruses of subgenotype VII (h and i) [6, 51].

The higher prevalence of AOAV-1 genotype VII.1.1 (with highly identical F protein sequence) indicated that ND remains enzootic despite the extensive use of live and inactivated vaccines to control the disease. Comparing to the NDV vaccine strains used in Egypt, the nucleotide identity of the six isolates of AOAV-1 genotype VII.1.1 varied from 82.8 to 86% with the [VG/GA strains (Avinew) vaccine and vaccines of genotype I were slightly more identical than those of genotype II, LaSota, clone 30 and Hitchner B1]. Roohani et al. [52] reported that NDV genotype VII strains were higher divergent from the genotypes I or II based vaccines. Also, Xue et al. [53] recorded that the phylogenetic analyses of 11 isolates of genotype VII NDV showed low amino acid similarity with the genotype I and II vaccine strains emphasizing the necessity of development of a genotype-matched vaccine. Interestingly, the nucleotide identity with the F protein sequence of genotype VII vaccine (KBNPC415R2L) used in Egypt ranged from 93.6 to 94.5%, indicating the probability of higher protection of this type of vaccine, having the advantage of decreasing the virus multiplication and shedding into the environment to reduce the spread of this devastating viral infection among poultry flock.

It had been established that homologous genotype vaccines are more effective in preventing virus replication and shedding after challenge than heterologous NDV genotype VII vaccine [52, 54, 55]. However, Sultan et al. [56] recommended the combined vaccination regime of inactivated genotype VII-matched and live genotype II vaccines for three times along the rearing period in commercial layers for better protection against clinical disease, mortality and viral shedding confirming the results of Sedeik et al. [57]. The aforementioned authors reported that the use of either inactivated genotype VII or II once alone induced the insufficient protection against clinical disease, mortality, and viral shedding in broiler chickens.

## Conclusions

Based on the partial and complete sequence of F gene and according to the updated classification of AOAV-1, we can conclude the high prevalence of AOAV-1 genotype VII.1.1 in the Egyptian chicken flocks despite the intensive vaccination with live and killed ND vaccines, as all the 16 isolates tested belonged to this genotype. Current vaccination strategies in the Egyptian chicken flocks don’t appear to control effectively AOAV-1 genotype VII.1.1, given the high prevalence of this genotype in the vaccinated birds surveyed here. Better vaccination targets are needed to control this genotype and reduce the spread of this devastating viral infection in Egyptian poultry flocks.

## Methods

### Sample collection

A total of 120 various poultry flocks or backyard household places (broiler, broiler breeder, commercial layer, native Saso and Balady chickens and pigeons) from 10 Egyptian provinces (El Beheira, Alexandria, El Gharbia, EL Qaliuobia, El Dakahlia, Kafr El Shiekh, El Menofia, El Giza, Marsa Mattrouh, and El Sharkia) were examined during 2015–2019 (Table 4). The locations were identified following consultation with farm owners who had contacted us to discuss their current situation. The household sources capacity started from 50 birds, while the organized commercial flocks’ capacity ranged from 3000 to 70.000 birds. The age of commercial broilers ranged from 18 to 36 days; layers: 52–315 days; Balady: 17–56 days; pigeons: 220–300 days; broiler breeders: 55–78 days and Saso: 45–55 days old.

**Table 4:** Sources of collected samples during 2016–2019

Total samples	Sample origin /(number)	Samples host /(number)	Types of Samples	Mortality within 1 week before sampling
120 from 10 provinces	El Behiera / (63) Alexandria/ (13) El Gharbia/ (15) El Qaliobia / (9) El Dakahlia / (7) Kafer El-Shiekh / (3) El Monofia / (3) El Giza/ (3) Marsa Mattrouh / (2) El Sharkia / (2)	Broiler / (90) Layers/ (13) Balady chickens/ (7) Pigeon / (4) Broiler Breeders / (3) Saso / (3)	Organs (trachea, lung, spleen, liver. Kidney, cecal tonsils) Brain (in case of nervous signs). Swabs (tracheal & cloacal)	10–35%

These examined poultry flocks suffered from high mortality ranged from 10 to 35% within the last week before sampling. Typical clinical signs and post-mortem lesions suspecting ND were seen before sampling. All flocks were vaccinated at least with live with or without inactivated vaccine regimens for NDV. Samples were collected from trachea, lung, spleen, kidney, liver, and brain tissues and/or swabs of sick and freshly dead birds. Samples were labeled and transported immediately on ice either to the laboratory of poultry and fish diseases department, Faculty of Veterinary Medicine Damanhour University, or Reference Laboratory for Veterinary Quality Control on Poultry production (RLQP) Biotechnology unit, Animal Health Research Institute. A single sample pool for each examined flock was processed except the brain tissues were processed separately according to the protocol of OIE [2].

### Virus isolation, propagation, and identification

Tissue homogenate and/or swabs from 3 to 5 birds/ each flock (as a pooled sample) was inoculated via allantoic sac of 3 Specific Pathogen Free Embryonated Chicken Eggs (SPF-ECE) 10 days old (Kom Oshem, SPF Farm, El Fayoum) according to OIE [2]. The harvested allantoic fluid of each sample was tested for haemagglutination activity by slide HA test after each passage [28].

### Extraction of viral RNA

Total viral RNA was extracted from all positive HA allantoic fluids using QIAamp Viral RNA Mini Kit (QIAGEN, Germany) according to manufacturer instructions**.**

### Detection of virulent AOAV-1 and co-infection with H5, H9, and IBV by quantitative or real-time reverse transcriptase polymerase chain reaction (qRT-PCR)

Oligonucleotide primers and probes used were supplied from Metabion; PCR reaction was conducted using real-time PCR (Step one Applied biosystems) according to manufacturer instructions. The oligonucleotide primers and probes used according to Wise et al. [58] and Creelan et al. [59] for NDV matrix and velogenic F gene, respectively; Lȍndt et al. [60] for highly pathogenic avian influenza (HPAI), H5; Ben Shabat et al. [61] for low pathogenic avian influenza (LPAI) H9 and Callison et al. [62] for infectious bronchitis virus (IBV).

### Gene sequencing of fusion (F) protein

Partial F gene sequencing for 10 AOAV-1 suspected isolates using primer sets designed by Selim et al. [27] was performed. Also, a full gene sequencing was carried on for the other six suspected AOAV-1 isolates rather than the above mentioned ten strains. Purification through QIAquick Gel Extraction Kit (Qiagen, Germany) and the one-step rRT-PCR reaction was used according to the manufacturer instructions. Sequence analysis using Big Dye Terminator V3.1 cycle sequencing kit (Perkinelmer, Foster city, CA) and Applied Biosystems 3130 genetic analyzer (ABI, USA) were done**.**

### Bioinformatic and phylogenetic analysis of F protein of AOAV-1

Relationships of the full F gene were compared with previously published NDV reference strains available in the public database (BLASTn, NCBI, USA) (http://www.ncbi.nlm.nih.gov/BLAST), using Bioedit software version 7.2.4. Nucleotides similarity and divergence were conducted in the MegAlign program of the Laser gene package (DNASTAR Inc., Madison, WI, USA). Molecular Evolutionary Genetics Analysis (MEGA) X version 2018 across computing platforms Software [63] was used in the construction of phylogenetic trees for the F gene nucleotide and amino acid sequences of the isolates. Algorithms to a matrix of pairwise distances estimated using the Maximum Composite Likelihood (MCL) approach and Tamura-Nei model [64].

### Pathogenicity evaluation

Intracerebral Pathogenicity Index (ICPI) was carried out for evaluation of the pathogenicity of 10 isolates of AOAV-1 from different provinces according to procedures of OIE [2].

## Data Availability

The datasets used and/or analysed during the current study are available from the corresponding author on reasonable request. All the sequences are registered on the NCBI database, and these are their links: AOAV1-Eg-Ch-18-2015, F, Complete, https://www.ncbi.nlm.nih.gov/nuccore/MH445410.1?report=GenBank; MH445410 AOAV1-Eg-Ch-B36-2017, F, Complete, https://www.ncbi.nlm.nih.gov/nuccore/MK984236.1?report=GenBank; MK984236 AOAV1-Eg-Ch-D30-2018, F, Complete, https://www.ncbi.nlm.nih.gov/nuccore/MK984237.1?report=GenBank; MK984237 AOAV1-Eg-Ch-R78-2018, F, Complete, https://www.ncbi.nlm.nih.gov/nuccore/MK984238.1?report=GenBank; MK984238 AOAV-Eg-Ch-F2-2016, F, Complete, https://www.ncbi.nlm.nih.gov/nuccore/MK984239.1?report=GenBank; MK984239 AOAV-Eg-Ch-MN51-2019, F, Complete, https://www.ncbi.nlm.nih.gov/nuccore/MN519684.1?report=GenBank; MN519684 NDV/Egypt/Ch/MR89/2017, F, Partial, https://www.ncbi.nlm.nih.gov/nuccore/MN519688.1?report=GenBank; MN519688 NDV/Egypt/Ch/MH6/2018 F, Partial, https://www.ncbi.nlm.nih.gov/nuccore/MN519690.1?report=GenBank; MN519690 NDV/Egypt/Ch/MDK3/2018 F, Partial, https://www.ncbi.nlm.nih.gov/nuccore/MN519692.1?report=GenBank; MN519692 NDV/Egypt/Ch/MQ2/2018 F, Partial, https://www.ncbi.nlm.nih.gov/nuccore/MN519696.1?report=GenBank; MN519696 NDV/Egypt/Ch/MQ3/2018, F, Partial, https://www.ncbi.nlm.nih.gov/nuccore/MN519693.1?report=GenBank; MN519693 NDV/Egypt/Ch/MGhl/2018, F, Partial, https://www.ncbi.nlm.nih.gov/nuccore/MN519689.1?report=GenBank; MN519689 NDV/Egypt/Ch/MK/2018, F, Partial, https://www.ncbi.nlm.nih.gov/nuccore/MN519694.1?report=GenBank; MN519694 NDV/Egypt/Ch/MQ4/2018, F, Partial, https://www.ncbi.nlm.nih.gov/nuccore/MN519687.1?report=GenBank; MN519687 NDV/Egypt/P/MR82/2018, F, Partial, https://www.ncbi.nlm.nih.gov/nuccore/MN519685.1?report=GenBank; MN519685 NDV/Egypt/P/MR84/2018, F, Partial, https://www.ncbi.nlm.nih.gov/nuccore/MN519686.1?report=GenBank.ncbi.nlm.nih.gov/nuccore/MN519686.1?report=GenBank; MN519686.

## Abbreviations

AOAV-1: Avian Orthoavulavirus-1
APMV-1: Avian paramyxoviruses 1
HPAI: Highly Pathogenic Avian Influenza
IBV: Infectious Bronchitis Virus
ICPI: Intracerebral Pathogenicity Index
LPAI: Pathogenic Avian Influenza
LPAI-H9: Low Pathogenic Avian Influenza
MCL: Maximum Composite Likelihood
MEGA: Molecular Evolutionary Genetics Analysis
ND: Newcastle disease
RLQP: Control on Poultry production
SPF-ECE: Specific Pathogen Free Embryonated Chicken Eggs
vNDV: Velogenic Newcastle disease virus
